# Real-world clinical treatment outcomes in Chinese non-small cell lung cancer with *EGFR* exon 20 insertion mutations

**DOI:** 10.3389/fonc.2022.949304

**Published:** 2022-09-02

**Authors:** Chao Shi, Ruyue Xing, Mengmeng Li, Junnan Feng, Rui Sun, Bing Wei, Yongjun Guo, Jie Ma, Huijuan Wang

**Affiliations:** ^1^ Department of Molecular Pathology, The Affiliated Cancer Hospital of Zhengzhou University, Henan Cancer Hospital, Zhengzhou, China; ^2^ Henan Key Laboratory of Molecular Pathology, Zhengzhou, China; ^3^ Department of Medical Oncology, The Affiliated Cancer Hospital of Zhengzhou University, Henan Cancer Hospital, Zhengzhou, China

**Keywords:** *EGFR* exon20 insertion, near loop, far loop, clinical treatment outcomes, NSCLC

## Abstract

**Background:**

*EGFR* exon 20 insertions (*EGFR* ex20ins) constitute a heterogeneous subset of *EGFR*-activating alterations. However, the effectiveness of standard therapy in patients with *EGFR* ex20ins remains poor.

**Methods:**

In our study, we retrospectively collected next-generation sequencing (NGS) data from 7,831 Chinese NSCLC patients and analyzed the relationship between *EGFR* ex20ins variations and medical records.

**Results:**

Our data showed that *EGFR* ex20ins account for up to 3.5% of all *EGFR* mutation non-small-cell lung cancer (NSCLC) patients and 1.6% of all NSCLC patients in China. Thirty-eight different variants of *EGFR* ex20ins were identified in 129 NSCLC patients. We observed that the patients with *EGFR* ex20ins may benefit from the anti-angiogenesis agents significantly (*P* = 0.027). In the *EGFR* ex20ins near-loop group, patients who received second-/third-generation EGFR-TKI therapy treatment as first-line treatment had a longer median progression-free survival (PFS) than those who initiated treatment with first-generation EGFR-TKI or chemotherapy. Patients with co-mutations of *EGFR* ex20ins near-loop and *TP53* tended to have a shorter OS in second-/third-generation EGFR-TKI therapy (*P* = 0.039). Additionally, median PFS was significantly longer in patients harboring *EGFR* ex20ins far-loop variants who received chemotherapy as a first-line setting (*P* = 0.037).

**Conclusions:**

Overall survival was significantly longer in *EGFR* ex20ins patients with anti-angiogenesis agents. For the choice of first-line strategy, NSCLC with *EGFR* ex20ins near-loop variants may benefit from second-/third-generation EGFR-TKI, while patients harboring *EGFR* ex20ins far-loop variants might have better outcomes from chemotherapy. *TP53* could serve as a potential predictive marker in poor prognosis for *EGFR* ex20ins near-loop patients.

## Introduction

The epidermal growth factor receptor (*EGFR*) gene is composed of 28 exons, which exist in the 7p21-14 region of the short arm of chromosome 7 with a length of 192 kbp. Most of these mutations occur between exons 18 and 21, and patients with these *EGFR* mutations respond to treatment with EGFR-tyrosine kinase inhibitors (EGFR-TKIs). Currently, *EGFR* mutation is the most widely studied in NSCLC. Exon 19 in-frame deletion and exon 21 L858R alterations are the two most sensitive *EGFR* mutations and are the two main mutant subtypes that respond best to TKI treatment (1, 2). *EGFR* exon 20 insertion mutation contains all the amino acid sites of exon 20 mutation translation and is 762-823, except for the classic drug resistance mutation T790M, which accounts for approximately 50% of all mutations. The frequency of *EGFR* exon 20 insertion (*EGFR* ex20ins) mutations in all NSCLC patients has been reported to range from 1% to 10% and approximately 4%–10% of *EGFR*-mutant NSCLC patients (3–5).

To date, a total of 85 unique *EGFR* ex20ins mutations have been identified in Chinese patients (6), while 64 unique *EGFR* ex20ins were found in the United States (7). Most *EGFR* ex20ins occur in Met766-Cys775 after the C-helix, and a few occur in Glu762-Tyr764 in the C-helix. The frequency of each *EGFR* ex20ins subtype is different. Previous studies have shown that *EGFR* A763_Y764insFQEA is sensitive to first-, second-, and third-generation drugs, suggesting that these patients have a better prognosis. However, data on the response of other *EGFR* ex20ins subtypes to treatment are still limited.

Studies have shown that most *EGFR* exon 20 point mutations were P-loop and αC-helix compressing (PACC) mutations, and *EGFR* exon 20 insertions occurring in the C-terminal loop of the αC-helix were considered to be a distinct subgroup: exon 20 loop insertions (Ex20ins-L). Ex20ins-L mutations could be subdivided into two subgroups: near-loop and far-loop Ex20ins (8).

Mobocertinib (9) and amivantamab (10) targeted therapy for *EGFR* ex20ins has been approved for clinical use; however, it remains an active area for drug development with several promising strategies currently investigated in clinical trials. The effectiveness of standard therapy in patients with *EGFR* ex20ins remains poor. Most of the currently approved agents and completed clinical studies have been in second-line and later settings, and it is necessary to investigate the effective first-line treatments for *EGFR* en20ins patients. A better understanding of the effectiveness of *EGFR* ex20ins standard therapy is needed to assess whether patients with different variants of *EGFR* ex20ins gain substantial benefit.

We attempted to describe patients’ clinical outcomes and responses to standard treatments. We used next-generation sequencing (NGS) to identify patients with *EGFR* ex20ins in our hospital and retrospectively evaluated their clinical outcomes, including different variation types and co-mutations.

## Materials and methods

### Study populations

**Figure 1:**
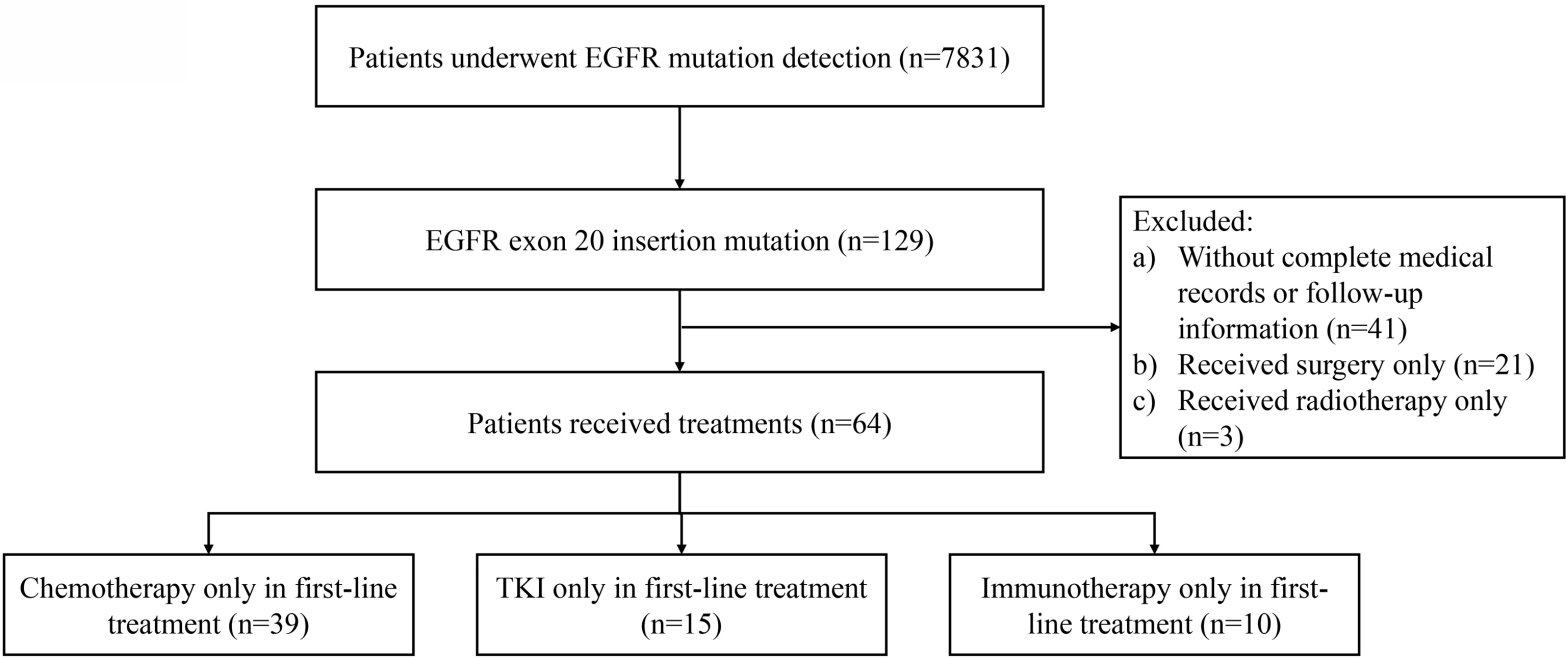
Flowchart of the study.

We conducted a single-center, retrospective study in Henan Cancer Hospital between 2016 and 2021. Genetic alterations were obtained from 7,831 tissue samples of NSCLC patients who underwent *EGFR* mutation screening by NGS. The inclusion criteria were as follows: (1) stage III/IV disease at initial diagnosis; (2) ≥18 years of age; (3) histologically or cytologically confirmed NSCLC; (4) *EGFR* ex20ins mutations confirmed at initial diagnosis by next-generation sequencing (NGS) with tumor tissues; (5) documented with available data of first-line therapies in medical records. 129 NSCLC patients with EGFR ex20ins were detected and 64 advanced NSCLC patients with EGFR ex20ins met the inclusion criteria and were further analyzed in this study (**Figure 1**). NSCLC histology was classified according to the World Health Organization standard (2018 Edition). All patients underwent clinical staging of lung cancer according to the TNM classification of the 7th International Association for the Study of Lung Cancer classification. Patients had to have a life expectancy of at least 3 months and were required to have a measurable disease per Response Evaluation Criteria in Solid Tumors (version 1.1). All procedures of this study were in accordance with the declaration of Helsinki (revised 2013). The study was approved by the Institutional Ethics Committee of Henan Cancer Hospital affiliated with Zhengzhou University (Approval number 2017407) and obtained the informed consent of all patients.

### Targeted NGS

DNA from formalin-fixed, paraffin-embedded tumor tissue samples was extracted. Comprehensive genomic profiling was performed by NGS with a 26- ((Novogene Co., Ltd., Beijing, China), 8- and 56- (Burning Rock Biotech, Guangzhou, China), and 1,021- (Geneplus Co., Ltd., Beijing, China) cancer-related gene panel covering the whole exons of the *EGFR* gene at a mean coverage depth of >800× (5,400 cases with 8 and 56 panels, 1,997 cases with 26 panels, and 434 cases with 1,021 panels). The genomic alterations including single base substitution, insertions/deletions, copy number variations, and gene rearrangement and fusions were assessed. Sequencing data were aligned to the human reference genome (build hg37) after removal of low-quality reads, using the BWA-MEM tool (v0.7.15) with default parameters (11). VarDict ((v1.4.6) (12) and VarScan (v2.4.2) (13) were utilized to call SNPs and small indels from the BAM files. Mutations were merged with those confirmed by both callers being selected as credible variants. The resulting variants were annotated by SnpEff (v4.3) (14) and then integrated into a unified database framework using Gemini (v0.19.1) (15).

### Immunohistochemistry

The primary antibodies used in this study include PD-L1 (22C3) (Dako, M3653, 1:50). The immunohistochemistry staining of PD-L1 (22C3) was performed on a Dako Autostainer Link 48 platform with the Dako K8002 detection kit, and the signals were amplified with a mouse linker (contained in the kit) and the signals enhanced with a DAB enhancer (Dako, S1961); the staining procedure was set similar to the FDA-approved PD-L1 (22C3) pharmDx staining procedure. All immunohistochemistry (IHC) sections were evaluated by two pathologists independently. PD-L1 ≥1% on tumor cells were defined as positive.

### Definition of *EGFR* ex20in helical region, near loop, and far loop

*EGFR* ex20ins helical region, near loop, and far loop were defined as follows. The *EGFR* ex20ins helical region was defined as the site on the αC-helix (E762–M766) of *EGFR* exon 20. *EGFR* ex20ins near loop was defined as the site on the loop following the αC-helix (A767–P772) of *EGFR* exon 20. *EGFR* ex20ins far loop was defined as the site on the loop following the αC-helix (H773–C775) of *EGFR* exon 20.

### Statistical analyses

The statistical analyses were performed using SPSS 20.0 (Chicago, IL, USA). Patients’ baseline characteristics were presented by descriptive statistics. A two-sided *P* value < 0.05 was considered statistically significant. The Kaplan–Meier method was adopted to estimate and plot the survival endpoints for progression-free survival (PFS), overall survival (OS), and differences between treatments were compared using the log-rank test.

## Results

### Patient characteristics

Patient characteristics are listed in **Table 1**. In all *EGFR* ex20ins patients, the median age was 56 years (range, 31 to 85 years), and 61 patients (47.3%) were men. Histologic examination detected that most were adenocarcinomas (80.6%), and 89 patients (69.8%) had stage IIIB and IV diseases. One hundred six cases were *EGFR* ex20ins near-loop patients, while the far-loop variants were 16 patients. Among them, 18.9% (n = 20) in near-loop variants and 43.8% (n = 7) in far-loop variants presented with a baseline central nervous system (CNS), respectively. Other clinicopathological characteristics, including T stage and site of metastasis, are shown in **Table 1**. Fifteen patients received EGFR-TKI treatment as a first-line treatment, and 39 patients received chemotherapy as a first-line strategy (**Figure 1**). The characteristics of the patients are listed in **Table 1**.

**Table 1:** Patient characteristics.

Characteristic	No. of *EGFR* ex20ins (N = 129)	No. of *EGFR* ex20ins near-loop (N = 106)	No. of *EGFR* ex20ins far-loop (N = 16)
Median age, years (range)	56 (31-85)	58 (31-85)	60 (42-79)
Sex			
Male	61 (47.3%)	49 (46.2%)	8 (50%)
Female	68 (52.7%)	57 (53.8%)	8 (50%)
Smoking history			
Never	101 (78.3%)	84 (79.2%)	12 (75%)
YES	28 (21.7%)	22 (20.8%)	4 (25%)
Drinking history			
Never	98 (76.0%)	80 (75.5%)	14 (87.5%)
YES	31 (24.0%)	26 (24.5%)	2 (12.5%)
Pathology			
Adenocarcinoma	104 (80.6%)	84 (79.2%)	13 (81.3%)
Squamous/adenosquamous	4 (3.1%)	2 (1.9%)	2 (12.5%)
Sarcomatoid	2 (1.6%)	2 (1.9%)	0
Unknown	9 (7.0%)	18 (17.0%)	1 (6.2%)
Stage			
I	5 (3.9%)	5 (4.7%)	0
II	8 (6.2%)	8 (7.5%)	0
III	9 (7.0%)	7 (6.6%)	1 (6.3%)
IV	81 (62.8%)	62 (58.5%)	13 (81.3%)
Unknown	26 (20.2%)	24 (22.7%)	2 (12.4%)
T stage			
T1-2	52 (40.3%)	46 (43.4%)	5 (31.3%)
T3-4	22 (17.1%)	17 (16.1%)	4 (25.1%)
Unknown	55 (42.6%)	43 (40.6%)	7 (43.8%)
Metastasis			
Yes	81 (62.8%)	62 (58.5%)	13 (81.2%)
No	36 (27.9%)	33 (31.1%)	2 (12.5%)
Unknown	12 (9.3%)	11 (10.4%)	1 (6.3%)
Site of metastasis			
None	36 (27.9%)	33 (31.1%)	2 (12.5%)
Brain	29 (22.5%)	20 (18.9%)	7 (43.8%)
Bone	38 (29.5%)	30 (28.3%)	5 (31.3%)
Other	14 (10.9%)	12 (11.3%)	1 (6.3%)
Unknown	12 (9.3%)	11 (10.4%)	1 (6.3%)

### Frequency and genetic characteristics of *EGFR* ex20ins mutations

**Figure 2:**
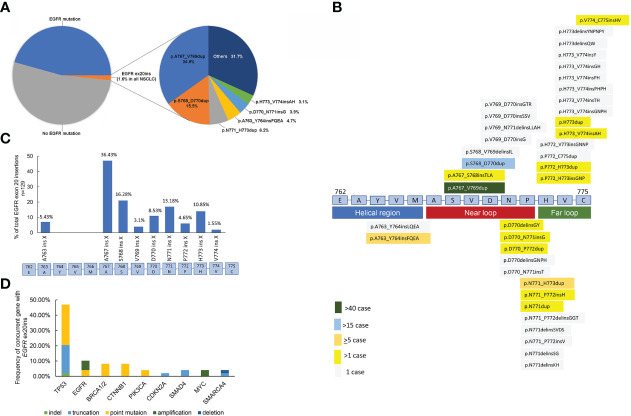
**(A)** Frequency of unique *EGFR* exon 20 insertions detected by comprehensive genomic profiling. Each alteration is shown as insertion (alternative nomenclature) or frequency. **(B)** Schematic of genomic positions of *EGFR* exon 20 insertions detected by comprehensive genomic profiling. **(C)** *EGFR* exon 20 insertion mutations in NSCLC. In particular, *EGFR* exon 20 mutations are in-frame insertions of between one and six amino acids (indicated as ins X) across a span of ~15 amino acids (E762–C775) in exon 20. The prevalence of exon 20 insertions that occur at different amino acid positions is shown by the blue bars. (n = 129). **(D)** Concurrent genomic alterations with *EGFR* exon 20 insertions in NSCLC.

Among the 7,831 unselective NSCLC tumors, our data showed that *EGFR* ex20ins mutations were detected in 129 patients, contributing 3.5% of all *EGFR*-mutation NSCLC and 1.6% of all NSCLC patients in China (**Figure 2A**). In total, 38 different variants of *EGFR* ex20ins were identified in 129 NSCLC patients. The most frequent variant was A767_V769dup (34.9%, 45/129), followed by S768_D770dup (15.5%, 20/129), N771_H773dup (8/129, 6.2%), A763_Y764insFQEA (6/129, 4.7%), D770_N771insG (5/129, 3.9%), and H773_V774insAH (4/129, 3.1%) (**Figure 2B**). Unique *EGFR* ex20ins mutations detected by NGS are summarized in **Figure 2B**. A767 (36.43%) and S768 (16.28%) were the most common insertion sites in *EGFR* in NSCLC (**Figure 2C**). After amino acid position 769 of the EGFR protein, these mutations are more heterogeneous at the molecular level, as in-frame insertions or duplications of between 3 and 18 bp (corresponding to 1–6 amino acids) clustered.

Putative cooccurring driver alterations in genes including *EGFR* (ex19del and L858R), erb-b2 receptor tyrosine kinase 2 (*HER2*), hepatocyte growth factor receptor gene (*MET*), and *KRAS* mutations tended to be mutually exclusive from *EGFR* ex20ins, and no concurrent *ALK*, *ROS1*, and *RET* fusions or *BRAF* mutations were identified. The most common concurrent alterations affected tumor protein p53 (*TP53*) (46.94%), *EGFR* (10.2%), breast cancer susceptibility gene (*BRCA*) (8.16%), and catenin beta 1 (*CTNNB1*) (8.16%) (**Figure 2D**).

To further determine whether *EGFR* ex20ins were associated with the expression of PD-L1, we classified our patients into two categories based on the expression of PD-L1-positive (≥1%) and PD-L1-negative (<1%). *EGFR* ex20ins variation mutations and insertion sites were negatively correlated with the expression of PD-L1 (not statistically significant). Interestingly, we observed that more cases were PD-L1-negative at amino acid position 773 of the EGFR protein (**Figure S1**).

### Antitumor activity of clinical treatments for patients with *EGFR* ex20ins

**Figure 3:**
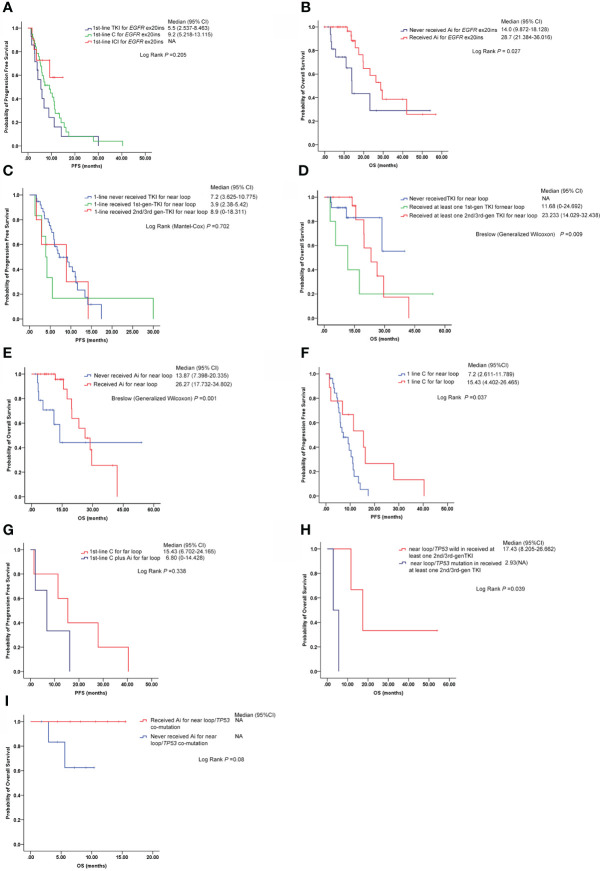
Responses to different therapies in different *EGFR* ex20ins variants (near loop and far loop). **(A)** Comparison of median PFS among first-line chemotherapy (chemotherapy, C), first-line EGFR*-*TKIs (all generations) (TKI) and first-line immunotherapy (ICI) in *EGFR* ex20ins NSCLC patients. **(B)** Median overall survival (mOS) time of two groups: the patients who never received anti-angiogenesis (Ai) therapy and the patients who received anti-angiogenesis therapy. **(C)** Median progression-free survival (mPFS) time of *EGFR* ex20ins near-loop variants under different EGFR-TKI treatments as first-line. **(D)** Median overall survival (mOS) time of *EGFR* ex20ins near-loop variants under different EGFR-TKI agents. **(E)** Comparison of the median overall survival (mOS) time between the group that never received anti-angiogenesis and the group that received anti-angiogenesis in *EGFR* ex20ins near-loop variants. **(F)** Median progression-free survival (mPFS) time of patients with *EGFR* ex20ins near-loop and far-loop variants on chemotherapy treatment as first-line. **(G)** In *EGFR* ex20ins far-loop variants, the mPFS between mono-chemotherapy and chemotherapy plus anti-angiogenesis was compared. **(H)** In the group with at least one second-/third-generation EGFR-TKI, the mPFS between *EGFR* ex20ins near-loop/*TP53*-wild and *EGFR* ex20ins near loop/*TP53*-mutation is shown. **(I)** Comparison of mOS between those who never received anti-angiogenesis treatments and those who received anti-angiogenesis agents in patients with *EGFR* ex20ins near loop/*TP53*-mutation. C, Chemotherapy; TKI, EGFR-TKIs; Ai, anti-angiogenesis; ICI, immunotherapy; NA, not available.

Thirty-nine *EGFR* ex20ins patients with stage IV lung adenocarcinoma started chemotherapy as first-line treatment, 15 patients received *EGFR*-TKI therapy as first-line therapy, and immunotherapy was administered as a first-line setting in 10 patients. The median PFS (mPFS) was 9.2 months (95% CI: 5.218–13.115) and 5.5 months (95% CI: 2.537–8.463) for patients who received chemotherapy and EGFR-TKI therapy, respectively (log-rank *P* = 0.205) (**Figure 3A**). A significant median OS difference between the group of chemotherapy or EGFR-TKI monotherapy and chemotherapy or TKI plus anti-angiogenesis (median, 14.0 *vs*. 28.7 months, log-rank *P* = 0.027) (**Figure 3B**) was observed.

### Clinical treatment outcomes for patients with *EGFR* ex20ins near loop

One hundred six patients with lung adenocarcinoma bore *EGFR* ex20ins near loop, six patients started first-generation *EGFR*-TKI therapy as first-line treatment, five patients received second-/third-generation *EGFR*-TKI therapy as first-line therapy, and chemotherapy or immunotherapy was administered as a first-line strategy in 37 patients. The median PFS (mPFS) of chemotherapy or immunotherapy, first-generation *EGFR*-TKI therapy, and second-/third-generation *EGFR*-TKI therapy as first line was 7.2 months (95% CI: 3.625–10.775), 3.9 months (95% CI: 2.380–5.420), and 8.9 months (95% CI: 0–18.311), respectively (log-rank *P* = 0.706) (**Figure 3C**). The patients who received at least one first-generation *EGFR*-TKI had a shorter median overall survival (mOS) (11.68 months, 95% CI: 0–24.692) than those who received at least one second-/third-generation *EGFR*-TKI (mOS: 23.233 months, 95% CI: 14.029–32.438), while the mOS of the group who never received *EGFR*-TKI was not available (Breslow (generalized Wilcoxon) *P* = 0.009) (**Figure 3D**). The median OS of patients with *EGFR* ex20ins near loop who received anti-angiogenic treatment was 26.27 months (95% CI: 17.732–34.802), significantly longer than in patients without anti-angiogenic treatment (13.87 months, 95% CI: 7.398–20.335) (Breslow (generalized Wilcoxon) *P* = 0.001) (**Figure 3E**).

### Clinical treatment outcomes for patients with *EGFR* ex20ins far loop

In this analysis, 27 patients with *EGFR* ex20ins near loop who received chemotherapy on first-line and 1st-line chemotherapy were included in eight NSCLC patients with *EGFR* ex20ins far loop, while the mPFS of patients with far loop was 15.43 months (95% CI: 4.402–26.465) longer than in the near-loop group which was 7.2 months (95% CI: 2.611–11.789) (log rank *P* = 0.037) (**Figure 3F**). The median PFS of five patients with *EGFR* ex20ins far loop on first-line mono-chemotherapy was 15.43 months (95% CI: 6.702–24.165); however, the mPFS of patients with first-line chemotherapy plus anti-angiogenesis was 6.80 months (95% CI: 0–14.428) (but no significant difference was noted) (**Figure 3G**).

### Impact of *EGFR* ex20ins mutations on immunotherapy

Among 10 patients who received immune checkpoint inhibitors (ICIs), three patients were treated with PD-1 or single-agent PD-L1 inhibitors, and the others were administered ICI plus pemetrexed/platinum chemotherapy. Per RECISIT 1.1, four (40%) patients achieved partial response (PR) and six patients (60%) obtained stable disease (SD).

Two patients with p. D770_P772dup who received sintilimab as the first-line treatment had very short mPFS (1.7 and 2.5 months). First-line ICIs were used in four patients whose EGFR protein was after amino acid position 770, resulting in short mPFS 1.7~3.8 months. The detailed outcomes of ICIs in *EGFR* ex20ins are listed in **Table 2**.

**Table 2:** Mutation characteristics and outcome of immunotherapy as first-line treatment.

Patient	Age	Gender	Mutations^a^ beforeICI (MAF)	ICI treatment	ICI drug	Antiangiogenic	Best response	PFS
1	51Y	F	p.N771_H773dup (5.06%)	ICI+ chemotherapy	Pembrolizumab	NO	SD	3.6m
2	85 Y	F	p.D770_P772dup (40.67%)	ICI	Sintilimab	NO	SD	2.5m
3	56 Y	M	p.A767_S768insTLA (20.1%)	ICI+ chemotherapy	Camrelizumab	YES	PR	14.8m
4	55 Y	M	p.A767_V769dup (47.4%)	ICI	Cemiolimab	NO	PR	11.4m
5	62 Y	M	p.S768_D770dup (30.38%)	ICI+ chemotherapy	Camrelizumab	YES	PR	10.6m
6	47 Y	F	p.H773dup (30.98%)	ICI+ chemotherapy	Sintilimab	YES	SD	3.8m
7	49 Y	M	p. A767_V769dup (19.6%)	ICI+ chemotherapy	Sintilimab	YES	PR	11.1m
8	47 Y	F	p.N771delinsSVDS (13.4%)	ICI+ chemotherapy	Pembrolizumab	NO	SD	9.1m
9	54 Y	M	p.A770_P772dup (43.2%)	ICI	Sintilimab	YES	SD	1.7m
10	47 Y	M	p.A767_V769dup (2.68%)	ICI+ chemotherapy	Pembrolizumab	YES	SD	13.0m

^a^ EGFR mutations

ICI, immune checkpoint inhibitor; MAF, mutation allele fraction; PFS, progression-free survival; PR, partial response; SD, stable disease; F, female; M, male.

### Treatment response for *EGFR* ex20ins with *TP53* co-mutation

We evaluated the frequency of concurrent genomic alterations. The most frequent concurrent mutation was *TP53* in the *EGFR* ex20ins cohort. In the group who received at least one second-/third-generation EGFR-TKI, patients with *EGFR* ex20ins near-loop/*TP53* mutations had a shorter mOS (2.93 months) than those with *EGFR* ex20ins near-loop/*TP53*-wild (17.43 months) (log-rank *P* = 0.039) (**Figure 3H**). Furthermore, in the *EGFR* ex20ins near-loop/*TP53* co-mutation subgroups, the patients with anti-angiogenesis treatment tended to show a longer mOS than those who did not (log-rank *P* = 0.08) (**Figure 3I**).

## Discussion

Our study evaluated the response of clinical treatments in advanced NSCLC patients with different *EGFR* ex20ins variant types (near loop and far loop). We noticed that the patients with the *EGFR* ex20ins far loop are more likely to have central nervous system metastases. Patients with *EGFR* ex20ins may benefit from the anti-angiogenesis therapy significantly. In the subgroup with *EGFR* ex20ins near loop, we observed a significant mPFS benefit from second-/third-generation TKI therapy as a first-line strategy compared with first-generation EGFR-TKI or chemotherapy. We also found that conventional chemotherapy as a first-line setting was achieved to improve PFS benefits for ex20ins far-loop patients. *TP53* could serve as a potential predictive marker in poor prognosis for *EGFR* ex20ins near-loop patients.

We collected 129 *EGFR* ex20ins NSCLC patients, which were all Chinese, contributing 3.5% of all *EGFR*-mutation NSCLC and 1.6% of all NSCLC patients. This result is consistent with the data reported in the literature, 4%–12% of *EGFR*-mutant NSCLC, and approximately 2% of all NSCLC (5, 7, 16, 17). Riess et al. and Qin et al. identified that the three most common molecular subtypes of *EGFR* ex20ins are A767_V769dup, S768_D770dup, and N771_H773dup, consistent with our conclusion, while more unique molecular subtypes were found in the US database (6, 7). The molecular subtypes are also different among distinct races. In our study, we reported 38 different variants of *EGFR* ex20ins in NSCLC. After the V769 insertion site, the types of *EGFR* ex20ins molecular variation were more diverse. Therefore, in the Chinese population, we suggest that the detection of *EGFR* ex20ins should include at least A767_V769dup, S768_D770dup, N771_H773dup, A763_Y764insFQEA, D770_N771insG, and H773_V774insAH. Alterations in *P53* were found in 46.94% of the samples and were very similar to the incidence of *P53* alterations in the US database (56%) (7). *EGFR* amplification was found in 10.2% of *EGFR* ex20ins patients, consistent with Yang et al. reporting 13.5% (18) and 22% of the US database (7).

In our study, the mPFS was not significantly different among the first-line chemotherapy group, the EGFR-TKI group, and immunotherapy group. However, another study reported that first-line conventional chemotherapy could improve PFS benefits for *EGFR* ex20ins patients compared with EGFR-TKI (18). We speculated that the reason for the inconsistent conclusions might be that most patients in the EGFR-TKI group received first-generation EGFR-TKIs in Yang’s study, while the patients in our study mainly used second- or third-generation EGFR-TKIs. We found out that the *EGFR* ex20ins patients treated with anti-angiogenesis might have a longer mOS in our study.

Recent studies have suggested that *EGFR* ex20ins mutations on A763_Y764, such as A763_Y764insFQEA patients, may be sensitive to EGFR-TKIs, while the other insertion variants are generally associated with insensitivity to available EGFR-TKIs. In recent years, clinical trials have reported novel EGFR-TKI or EGFR monoclonal antibodies, such as afatinib and cetuximab (NCT03727724) (19), erlotinib and cetuximab (NCT00895362) (20), osimertinib (NCT03414814) (21), poziotinib (22), luminespib (a HSP90 inhibitor) (23), TAS6417 (a novel EGFR TKI) (24), and TAK-788 (NCT02716116) (9, 25). However, these studies did not distinguish different *EGFR* ex20ins mutations. Both Fang et al. and Piotrowska et al. reported that osimertinib could effectively inhibit *EGFR* ex20ins in sporadic patients (26, 27), especially on p. S768_D770dup, p. A767_V769dup, p. N771_P772insL, p. D770_N771insG, and p. A763_Y764insFQEA mutations. Qin et al. reported that more than half of patients were treated with osimertinib after first-line TKI treatment, and patients with p. N771_P772insHN, p. S768_D770dup, p. A763_ Y764insFQEA, p. N771_ H773dup, or p. A767_V769dup had effective disease control (6). We found that the mPFS of the patients with *EGFR* ex20ins near-loop variants received second-/third-generation EGFR-TKIs as the first-line treatment for up to 8.9 months. Outcomes of different first-line strategies were observed in our study. Second-/third-generation EGFR-TKI therapy generated a superior clinical treatment efficacy for *EGFR* ex20ins near-loop patients compared with those taking first-generation TKI or treated with non-TKI therapies.

Previous studies revealed that structural analysis of *EGFR* ex20ins offers insight into the mechanism of different TKI responses. The crystal structure of *EGFR* p. D770_N771insNPG suggests that the insertion leads to the constitutive activation of *EGFR* by blocking the conformational rearrangements required for the inactive conformation of the kinase without increasing the binding affinity to EGFR-TKIs (28–30). In our study, the patients with near-loop variants of *EGFR* ex20ins who received at least one second-/third-generation EGFR-TKI had a longer mOS than other therapy treatments, while those patients with far-loop variants underwent chemotherapy as the first-line treatment had a longer mPFS than those who received TKI treatment. We found that patients with near-loop variants might benefit more from second-/third-generation EGFR-TKI. We speculated that this might also be related to the crystal structure of *EGFR* ex20ins. Chemotherapy was preferred as the first-line therapy for patients carrying *EGFR* ex20ins far-loop variation due to the longer PFS observed in our study.

In this study, 10 patients received treatment with immune checkpoint inhibitors (ICIs) alone or in combination with other agents. Due to the small sample size, the effect of ICIs in lung cancer patients harboring *EGFR* exon 20 insertion mutations is lacking. Nong et al. reported a case report of the clinical benefit of upfront immune checkpoint inhibitors (ICIs) plus chemotherapy for a brain metastatic NSCLC patient harboring *EGFR* exon 20 insertion mutation (31). However, it is still not clear whether our 10 patients with *EGFR* exon 20ins mutations can benefit from immune checkpoint inhibitors.

Yang et al. suggested that there was no significant difference in median PFS between first-line chemotherapy with bevacizumab and chemotherapy without bevacizumab in *EGFR* ex20ins patients (18). However, other studies indicated that the addition of anti-angiogenesis to TKIs improved median PFS compared to EGFR-TKIs alone in *EGFR*-mutant NSCLC (32–34). In our analysis, patients with *EGFR* ex20ins mutant NSCLC appear to benefit from the use of anti-angiogenesis agents on median OS, especially in the patients bearing *EGFR* ex20ins near-loop variants.

*TP53* mutations were found in 46% of the samples in our study and were very similar to the incidence of *P53* alterations in the Foundation Medicine database (56%) (7) and Noura’s data (48%) (48%) (35). *EGFR* amplification was found in 10% of the ex20ins patients and 13.5% of the patients in the Yang et al. database, while it was found in 22% of patients in the US database (7, 18). *EGFR* ex20ins near-loop patients with *TP53* co-mutation may have a shorter median OS; therefore, we speculated that *TP53* may be a poor prognostic factor for *EGFR* ex20ins near-loop variation patients.

The limitations of this study are that it was a retrospective, single-center study; the treatment comparison among chemotherapy, TKIs, or immunotherapy was not randomized and was based on patient factors (presentation status, socioeconomic status for patients who had to pay for chemotherapy and TKI) and physician factors (training, experience); the time span is relatively large, and the treatment plan is constantly being optimized and improved with the progress of research; and there must be bias in this process.

## Conclusions

Our study showed that angiogenesis inhibitors might yield a better survival benefit in advanced NSCLC with *EGFR* ex20ins. Second-/third-generation EGFR-TKI therapy as first-line therapy might improve PFS benefits for ex20ins near-loop patients than chemotherapy alone or first-generation EGFR-TKI. *TP53* could serve as a potential predictive marker in poor prognosis for this subset of patients. *EGFR* ex20ins far-loop patients gave priority to chemotherapy as a first-line setting, which may bring longer PFS. It is necessary to detect *EGFR* ex20ins variation accurately for the choice of clinical strategy.

## Data availability statement

The datasets presented in this study can be found in Genome Sequence Archive (GSA) database with the accession numbers of HRA002728, further inquiries can be directed to the corresponding author.

## Ethics statement

This study was reviewed and approved by Institutional Ethics Committee of Henan Cancer Hospital affiliated with Zhengzhou University. The patients/participants provided their written informed consent to participate in this study. Written informed consent was obtained from the individual(s) for the publication of any potentially identifiable images or data included in this article.

## Author contributions

(1) Conception and design: HW and JM. (2) Administrative support: HW and JM. (3) Provision of study materials or patients: CS and RX. (4) Collection and assembly of data: RX, ML, JF, RS, and BW. (5) Data analysis and interpretation: CS and RX. (6) Manuscript writing: CS. (7) Supervision: HW, JM, and YG. (8) Editing and review of writing: HW and JM. (9) All authors contributed to the article and approved the submitted version.

## Funding

This work was supported by the National Natural Science Foundation of China (grant number 81802444), Science and Technology Department of Henan Province (grant number 212102310128), Medical Science and Technology Project of Henan Province (grant number: SBGJ202103033), and Major Public Welfare Projects in Henan Province (grant number 201300310400) - Research and Development of New Technologies for Tumor Liquid Biopsy and Immunotherapy.

## Conflict of interest

The authors declare that the research was conducted in the absence of any commercial or financial relationships that could be construed as a potential conflict of interest.

## Publisher’s note

All claims expressed in this article are solely those of the authors and do not necessarily represent those of their affiliated organizations, or those of the publisher, the editors and the reviewers. Any product that may be evaluated in this article, or claim that may be made by its manufacturer, is not guaranteed or endorsed by the publisher.

## Supplementary material

Supplementary Figure 1 Correlation between expression of PD‐L1 and each insertion site of *EGFR* ex20ins.

## Abbreviations

Epidermal growth factor receptor, *EGFR*; *EGFR* exon20-insertions, *EGFR* ex20ins; Tyrosine kinase inhibitors, TKIs; Next-generation sequencing, NGS; Progression-free survival, PFS; Formalin-fixed paraffin-embedded, FFPE; Non-small cell lung cancer, NSCLC; Overall survival, OS.
