# Supplementary figures and images for: Real-world clinical treatment outcomes in Chinese non-small cell lung cancer with EGFR exon 20 insertion mutations
Source: Front Oncol. 2022 Sep 2;12:949304. doi: 10.3389/fonc.2022.949304 (PMC9479138; doi:10.3389/fonc.2022.949304)

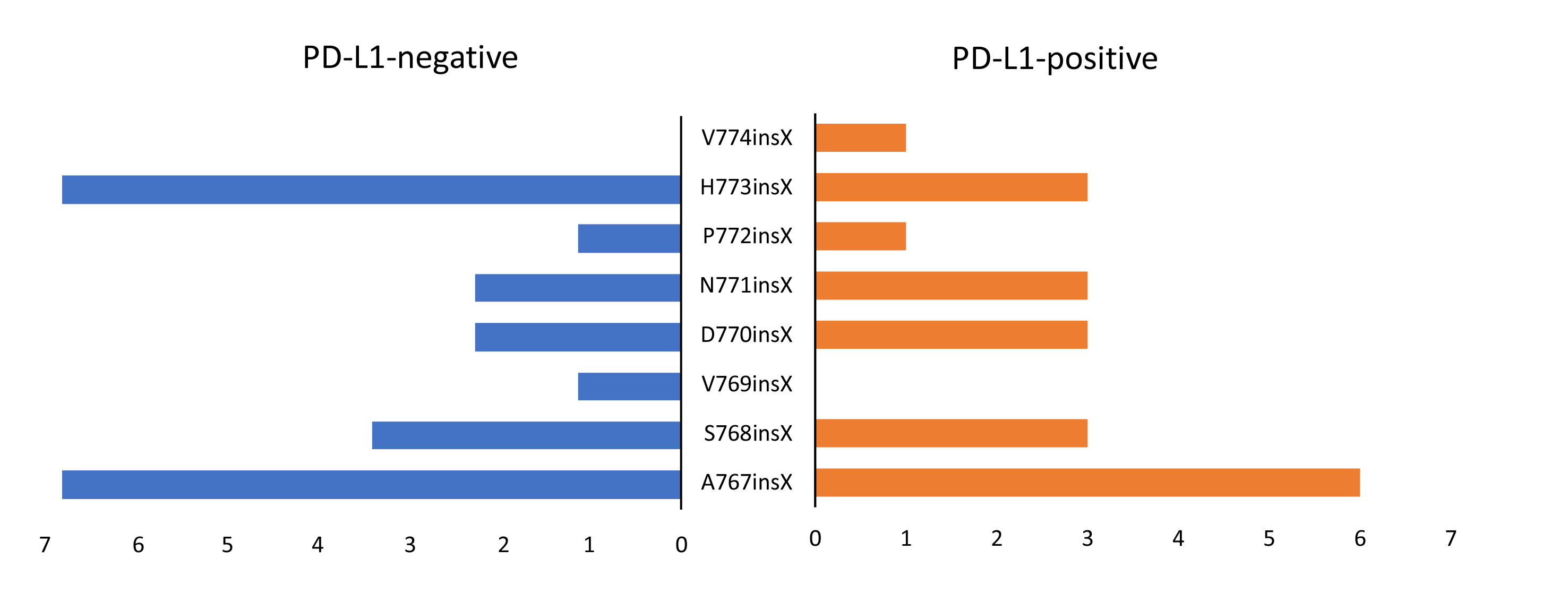

Supplement: Supplementary Figure 1 — Correlation between expression of PD‐L1 and each insertion site of EGFR ex20ins. [file Image_1.jpeg]
